# Clinical characteristics, management, and outcomes of nivolumab-associated pituitary toxicity: a pooled analysis of published case reports and case series

**DOI:** 10.3389/fendo.2026.1929591

**Published:** 2026-10-21

**Authors:** Zheng Liu, Nan Huang, Ronghui Li, Haibo Lei, Xiang Liu

**Affiliations:** Clinical pharmacy, The Central Hospital of Xiangtan (The Affiliated Hospital of Hunan University), Xiangtan, China

**Keywords:** hypophysitis, isolated acth deficiency, nivolumab, pooled analysis, secondary adrenal insufficiency

## Abstract

**Objective:**

To summarize the clinical features, diagnosis, management, and outcomes of nivolumab-associated pituitary toxicity.

**Methods:**

We conducted a pooled analysis of published case reports and case series describing nivolumab-associated pituitary toxicity. PubMed, Embase, Web of Science Core Collection, CNKI, and Wanfang Data were searched from inception to June 8, 2026, supplemented by reference-list screening. Reports with extractable individual patient data describing nivolumab-associated hypophysitis, isolated adrenocorticotropic hormone (ACTH) deficiency, or central/secondary adrenal insufficiency were included. Combination immune checkpoint inhibitor therapy, insufficient data, and duplicate reports were excluded. Data were extracted per patient. Continuous and categorical variables are presented as median (range) and n/N (%), respectively.

**Results:**

Forty-five independent patients from 43 reports were included; 33 (73.3%) were male, and median age was 68 years (39–85). Toxicity occurred after a median of 9 nivolumab cycles (n=40; range, 1–33); 8 patients (17.8%) developed symptoms after discontinuation, with a median delay of 24 weeks (4–56). Fatigue (41/44, 93.2%), anorexia (32/45, 71.1%), and nausea/vomiting (23/44, 52.3%) predominated. Hyponatremia occurred in 24/33 patients (72.7%). ACTH was low in 38/44 patients (86.4%) and inappropriately normal in 6/44 (13.6%) despite low cortisol; basal cortisol was reduced in 43/44 (97.7%). Pituitary MRI abnormalities were reported in 11/41 patients (26.8%). Hydrocortisone was the most frequently used replacement therapy, and symptoms improved in 44/45 patients (97.8%). Among 29 patients with hormonal follow-up, 2 (6.9%) had partial ACTH–cortisol axis recovery; none had complete recovery. No recurrence was reported among six patients rechallenged with nivolumab.

**Conclusion:**

Nivolumab-associated pituitary toxicity mainly affects the ACTH–cortisol axis and may present without MRI abnormalities. Prompt assessment of cortisol, ACTH, and electrolytes is warranted in symptomatic patients.

## 1 Introduction

Nivolumab is a monoclonal antibody targeting programmed cell death-1 (PD-1). By blocking the inhibitory signals of PD-1 and PD-L1/PD-L2, nivolumab can enhance the T-cell-mediated anti-tumor response and has shown clinical benefits in the treatment of various malignant tumors such as melanoma, non-small cell lung cancer, renal cell carcinoma, and esophageal cancer (1–4). However, extensive immune activation resulting from the release of immune checkpoints may also lead to immune-related adverse events (irAEs), involving multiple organ systems such as the skin, digestive, respiratory, nervous and endocrine systems (5, 6). The clinical manifestations of endocrine irAEs are often insidious. However, if adrenal insufficiency is missed, it may rapidly progress to circulatory failure, hypoglycemia, hyponatremia and even adrenal crisis (7–9).

Previous studies have shown that the clinical manifestations of ICI-related pituitary toxicity vary significantly depending on the type of drug. The incidence of hypophysitis caused by anti-cytotoxic T-lymphocyte-associated protein 4 (CTLA-4) inhibitors (such as ipilimumab) is relatively high (1% - 17%), usually presenting as diffuse pituitary enlargement. It involves multiple pituitary axes, including the thyroid stimulating hormone (TSH), gonadotropin (FSH/LH), and adrenocorticotropic hormone (ACTH) axes (10, 11). The pathogenesis is closely related to the expression of CTLA-4 on the surface of pituitary cells and the complement-dependent cytotoxic effects resulting therefrom (12). In contrast, the incidence of pituitary toxicity associated with anti-PD-1 monotherapy is usually lower, previously reported to be approximately 0.5% to 1.5%. Its clinical manifestations are often mainly the involvement of the ACTH-cortisol axis, and headache and pituitary MRI abnormalities are relatively rare (13, 14). Anti-PD-1-associated pituitary toxicity often presents as isolated ACTH deficiency (IAD). In anti-PD-1-associated pituitary toxicity, pituitary MRI usually shows no obvious abnormalities, and the clinical manifestations are insidious, which is easy to be ignored or delayed in diagnosis (13–15).

Due to the relatively low incidence of pituitary toxicity induced by nivolumab, there is currently a lack of systematic studies with large sample sizes. Most of the previous literature is case reports or small sample case series, and the understanding of the clinical characteristics, optimal treatment strategies and long-term prognosis of such adverse reactions is still insufficient. Therefore, this study identified and pooled published individual cases of nivolumab-associated pituitary toxicity to characterize their clinical manifestations, laboratory and imaging findings, treatment patterns, and outcomes.

## 2 Data and methods

### 2.1 Study design and case source

This study was a descriptive pooled analysis based on publicly available individual case data. PubMed, Embase, Web of Science Core Collection, CNKI, and Wanfang Data were searched from inception to June 8, 2026. The search strategy combined terms related to nivolumab and pituitary toxicity using the Boolean operators and OR. Free-text terms were used, including “nivolumab,” “Opdivo,” “BMS-936558,” “hypophysitis,” “hypopituitarism,” “isolated ACTH deficiency,” “ACTH deficiency,” “secondary adrenal insufficiency,” “central adrenal insufficiency,” and “pituitary dysfunction.” In PubMed, these terms were searched in the Title/Abstract fields; no additional MeSH terms were used. The complete database-specific search strategies are provided in **Supplementary Table S1**. The language limit was English. Reference lists of eligible articles and relevant reviews were additionally screened manually. The initial search obtained 2592 records. After deduplication, potentially relevant records were screened manually, full texts were assessed for eligibility, and patient-level deduplication was performed. Ultimately, 45 independent patients from 43 reports were included (**Figure 1**).

**Figure 1:**
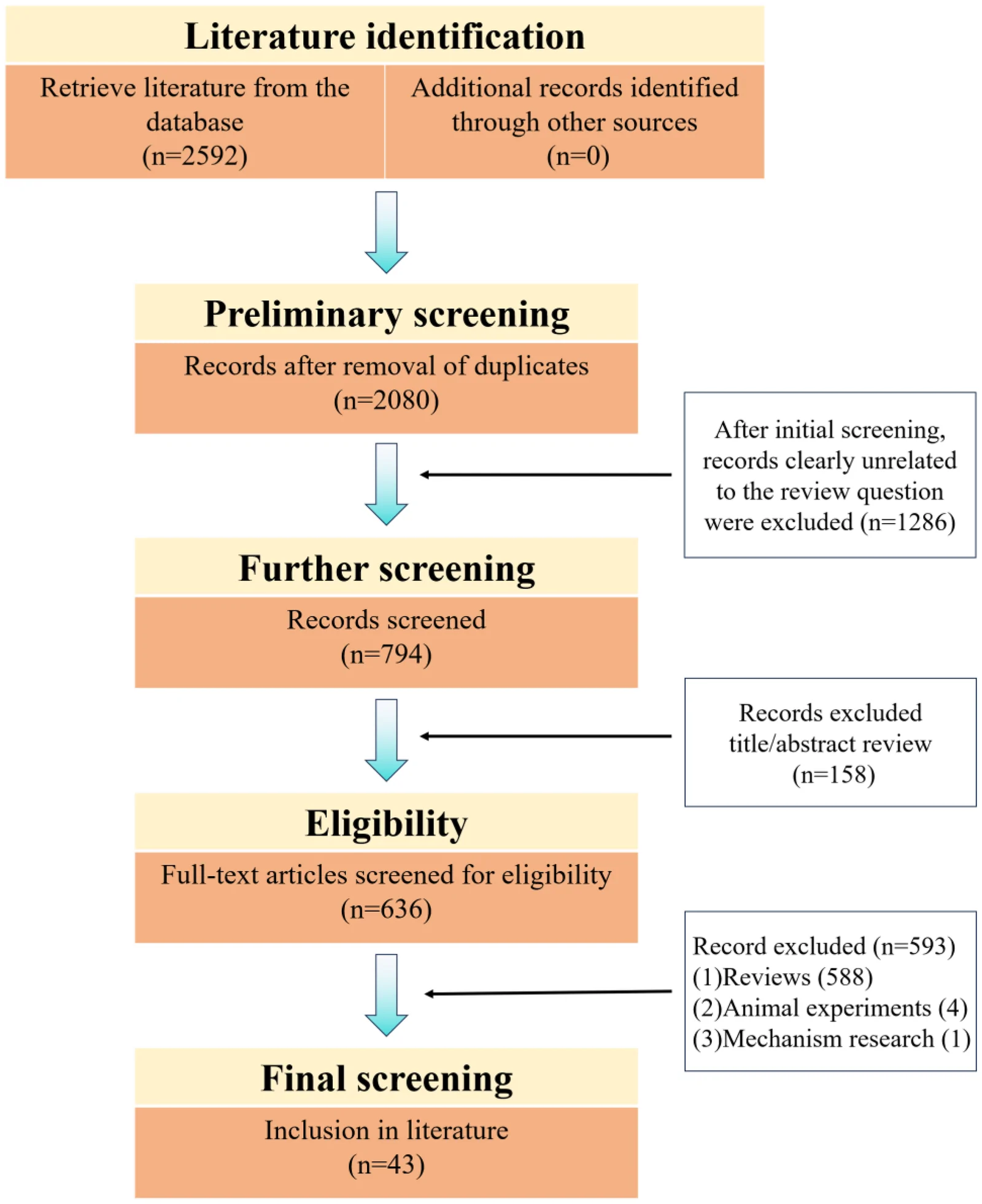
Flow chart of literature screening.

### 2.2 Inclusion and exclusion criteria

Inclusion criteria: (1) Case reports or case series; (2) The patient received nivolumab monotherapy; (3) The original report attributed hypophysitis, isolated ACTH deficiency, central/secondary adrenal insufficiency, or another relevant pituitary abnormality to nivolumab; and (4) sufficient objective endocrine information was available to support pituitary–adrenal dysfunction. For cases included on the basis of central/secondary adrenal insufficiency, author attribution alone was not considered sufficient; compatible biochemical evidence, such as low cortisol with low or inappropriately normal ACTH or an abnormal dynamic HPA-axis response, was required.

Exclusion criteria were: (1) nivolumab administered in combination with other immune checkpoint inhibitors when attribution to nivolumab could not be distinguished; (2) reviews, mechanistic studies, animal studies, or conference abstracts without individual patient data; (3) cases without documented nivolumab exposure or without sufficient information to verify the pituitary-related diagnosis; and (4) duplicate reports of the same patient. Potential duplicate patients across publications were identified by comparing available patient characteristics, including age, sex, tumor type, country or institution, nivolumab exposure, timing of toxicity onset, clinical presentation, and key laboratory findings. When multiple publications were judged to describe the same patient, the report containing the most complete clinical, diagnostic, treatment, and follow-up information was retained.

### 2.3 Definition of pituitary toxicity and diagnostic phenotypes

In this study, “nivolumab-associated pituitary toxicity” was used as an operational umbrella term rather than as a single diagnostic entity. It encompassed published cases attributed to nivolumab in which dysfunction of the pituitary–adrenal axis was diagnosed as hypophysitis, isolated ACTH deficiency (IAD), or central/secondary adrenal insufficiency. These conditions were pooled because the terminology used across published case reports was heterogeneous and frequently overlapping, while the common clinical feature relevant to this analysis was nivolumab-associated impairment of the hypothalamic–pituitary–adrenal axis.

For case classification, hypophysitis was defined according to the diagnosis reported by the original authors, based on compatible clinical and endocrine findings, with or without pituitary MRI abnormalities. IAD referred to ACTH deficiency with cortisol deficiency in the absence of documented clinically relevant dysfunction of other pituitary hormonal axes, where sufficient information was available. Central/secondary adrenal insufficiency referred to cortisol deficiency accompanied by low or inappropriately normal ACTH, consistent with central impairment of the hypothalamic–pituitary–adrenal axis. Because the completeness of endocrine testing varied across reports, these categories were not considered mutually exclusive when the original publication used overlapping terminology. Throughout the manuscript, “nivolumab-associated pituitary toxicity” is used as the overarching term, whereas “hypophysitis,” “isolated ACTH deficiency (IAD),” and “central/secondary adrenal insufficiency” are used only when referring to specific diagnostic phenotypes.

### 2.4 Data extraction

Demographic characteristics, including age, sex, country/region, tumor type, and pre-existing endocrine or hormone-related history, were extracted. Additional variables included nivolumab exposure, cycles at onset, delayed onset after treatment discontinuation, symptoms and signs, serum sodium, ACTH, cortisol, results of dynamic hypothalamic–pituitary–adrenal (HPA) axis testing, pituitary MRI findings, initial and maintenance glucocorticoid therapy, nivolumab management, and follow-up outcomes. Data were collected using a structured case-report form.

### 2.5 Statistical analysis

Continuous variables were summarized as median (range), and categorical variables as n/N (%). Only descriptive statistics were performed. Incidence rates were not calculated, and no between-group comparisons, risk-factor analyses, or causal inferences were undertaken. Data management and descriptive analyses were performed using Microsoft Excel.

## 3 Results

### 3.1 Baseline characteristics of patients

Finally, 45 independent patients in 43 reports were included (**Table 1**). Of the 45 patients, 33 (73.3%) were male and 12 (26.7%) were female; the median age was 68 years (range, 39–85 years). The cases were mainly from Japan (26/45, 57.8%), followed by the United States (5/45, 11.1%) and China (4/45, 8.9%). Lung cancer and melanoma were the most common primary tumors, with 16 cases in each group (35.6%), followed by renal cell carcinoma in 7 cases (15.6%). 14 cases (31.1%) reported pre-existing endocrine- or hormone-related history, and 8 cases (17.8%) had thyroid, adrenal, Carney syndrome or cortisol related history. The previous medical history mentioned above is only a case description and should not be interpreted as risk factors for nivolumab-associated pituitary toxicity.

**Table 1:** Clinical characteristics of 45 patients with nivolumab-associated pituitary toxicity.

Parameter	Classification	Value
Sex	Male	33/45 (73.3%)
	Female	12/45 (26.7%)
Age, years	Median (range)	68 (39-85)
Country/region	Japan	26/45 (57.8%)
	United States	5/45 (11.1%)
	China	4/45 (8.9%)
	Turkey	2/45 (4.4%)
	India,	1/45 (2.2%)
	Germany	1/45 (2.2%)
	France	1/45 (2.2%)
	Italy	1/45 (2.2%)
	Morocco	1/45 (2.2%)
	Greece	1/45 (2.2%)
	Portugal	1/45 (2.2%)
	Belgium	1/45 (2.2%)
Primary cancer	Lung cancer	16/45 (35.6%)
	Melanoma	16/45 (35.6%)
	Renal cell carcinoma	7/45 (15.6%)
	Esophageal cancer	2/45 (4.4%)
	Other cancers (gastric cancer, n=3; mesothelioma, n=1)	4/45 (8.9%)
Pre-existing history	Any endocrine/hormone-related history*	14/45 (31.1%)
	Thyroid/adrenal/Carney/cortisol-related history*	8/45 (17.8%)
Time to onset	Cycles at onset, median (range); n=40	9 (1-33)
	Late onset after treatment cessation, median (range); n=8	24 (4-56) weeks

*These conditions were recorded descriptively and were not evaluated as risk factors. The endocrine/hormone-related history category included thyroid/adrenal conditions, diabetes mellitus, SIADH, or other relevant history as recorded in the case reports.

### 3.2 Onset time and clinical manifestations

Among the 40 patients with clear medication cycle data, pituitary toxicity occurred after a median of 9 cycles (ranging from 1 to 33 cycles) of nivolumab treatment. Eight patients (17.8%) developed symptoms or were diagnosed after discontinuation of nivolumab, with a median interval of 24 weeks (4 to 56 weeks) from the last administration. Fatigue was the most common (41/44, 93.2%), followed by anorexia (32/45, 71.1%), nausea/vomiting (23/44, 52.3%), and hypotension, syncope or relative hypotension (21/44, 47.7%); Headache (2/44, 4.5%) and polyuria (1/44, 2.3%) were less common (**Table 2**).

**Table 2:** Clinical presentation, laboratory findings, dynamic HPA-axis testing, and pituitary MRI features.

Parameter	Classification	Value
Symptoms and signs	Fatigue	41/44 (93.2%)
	Loss of appetite	32/45 (71.1%)
	Nausea/vomiting	23/44 (52.3%)
	Hypotension/syncope/relative hypotension	21/44 (47.7%)
	Headache	2/44 (4.5%)
	Polyuria	1/44 (2.3%)
Laboratory findings	Hyponatremia (<135 mEq/L or explicitly reported); n=33	24/33 (72.7%)
	ACTH low; n=44	38/44 (86.4%)
	ACTH inappropriately normal with low cortisol; n=44	6/44 (13.6%)
	Cortisol low; n=44	43/44 (97.7%)
	Cortisol not low at baseline but inadequate stimulated response; n=44	1/44 (2.3%)
Dynamic HPA-axis testing	Any reported abnormal HPA-axis response; n=27	27/27 (100.0%)
Pituitary MRI	Any pituitary MRI abnormality; n=41	11/41 (26.8%)
	No clear pituitary MRI abnormality; n=41	30/41 (73.2%)
	Pituitary enlargement/fullness; n=40	6/40 (15.0%)
	Stalk thickening; n=38	5/38 (13.2%)
	Partial empty sella; n=40	2/40 (5.0%)

Denominators exclude missing/uninterpretable fields. Dynamic tests included heterogeneous protocols (CRH stimulation, rapid ACTH/cosyntropin testing, insulin tolerance testing, or combined pituitary tests); this row indicates reported HPA-axis abnormality and does not establish a uniform anatomical localization. MRI findings are not mutually exclusive.

### 3.3 Laboratory tests, dynamic tests and pituitary MRI

Serum sodium data were available for 33 patients, of whom 24 (72.7%) had hyponatremia. ACTH data were available for 44 patients: ACTH was low in 38 (86.4%) and inappropriately normal despite low cortisol in 6 (13.6%). Basal cortisol data were available for 44 patients, of whom 43 (97.7%) had low levels. In the remaining patient, basal cortisol was not low, but the response to stimulation was inadequate. Dynamic HPA-axis testing was reported in 27 patients, all of whom showed abnormal responses. Given the heterogeneity of testing protocols, this finding should be interpreted only as evidence of HPA-axis dysfunction rather than as indicating a uniform physiological abnormality.

Among 41 patients with interpretable pituitary MRI findings, abnormalities were reported in 11 (26.8%), while 30 cases (73.2%) showed no definite abnormality. The specific manifestations included pituitary enlargement/fullness in 6/40 cases (15.0%), thickening of the pituitary stalk in 5/38 cases (13.2%), and partial empty sella turcica in 2/40 cases (5.0%). Multiple imaging manifestations can be combined in the same patient (**Table 2**).

### 3.4 Glucocorticoid therapy and nivolumab treatment

Initial glucocorticoid treatment regimens were recorded in 43 cases. Initial glucocorticoid therapy was documented in 43 patients. Treatment was initiated orally in 21 patients (48.8%) and intravenously in 16 (37.2%); the route was unclear or mixed in 6 (14.0%). Hydrocortisone was the most commonly used initial glucocorticoid (32/43, 74.4%), followed by prednisone/prednisolone (5/43, 11.6%) and methylprednisolone (4/43, 9.3%). Maintenance glucocorticoid replacement regimens were recorded in 39 cases, among which hydrocortisone was the most commonly used (32/39, 82.1%), followed by prednisone (6/39, 15.4%) and cortisone acetate (1/39, 2.6%) (**Table 3**).

**Table 3:** Reported glucocorticoid treatment, nivolumab management, and clinical outcomes.

Domain	Classification	Value
Initial glucocorticoid treatment (n=43)	Oral initiation	21/43 (48.8%)
	Intravenous initiation	16/43 (37.2%)
	Route unclear/mixed	6/43 (14.0%)
	Hydrocortisone	32/43 (74.4%)
	Prednisone/prednisolone	5/43 (11.6%)
	Methylprednisolone	4/43 (9.3%)
Maintenance glucocorticoid replacement (n=39)	Hydrocortisone	32/39 (82.1%)
	Prednisone	6/39 (15.4%)
	Cortisone acetate	1/39 (2.6%)
Nivolumab management	Continued	15/45 (33.3%)
	Temporary interruption	2/45 (4.4%)
	Stopped before or after toxicity	25/45 (55.6%)
	Unreported/indeterminate	3/45 (6.7%)
	Re-challenge	6/27 (22.2%)
Outcomes	Symptoms improved	44/45 (97.8%)
	Partial ACTH/cortisol recovery documented	2/29 (6.9%)
	No ACTH/cortisol recovery	27/29 (93.1%)
	Re-challenge: recurrence	0/6 (0%)
	Re-challenge: no recurrence	6/6 (100%)
	Alive at last reported follow-up	39/43 (90.7%)
	Deceased at last reported follow-up	4/43 (9.3%)

Treatment categories reflect case-report documentation and are not comparative efficacy data. “Stopped” includes cases in which nivolumab had been discontinued or completed before onset; the category should not be interpreted as discontinuation caused by pituitary toxicity. Later exposure/rechallenge definitions and follow-up were heterogeneous.

After the occurrence of pituitary toxicity, 15 cases (33.3%) continued to use nivolumab, 2 cases (4.4%) temporarily discontinued the drug, 25 cases (55.6%) had stopped treatment before or after the occurrence of toxicity, and the treatment methods of 3 cases (6.7%) were not reported or could not be determined. Six patients were rechallenged with nivolumab, and no recurrence of pituitary toxicity was reported. However, the definition of rechallenge, duration of follow-up, oncologic context, and hormone replacement strategies varied across reports.

### 3.5 Clinical outcomes

After glucocorticoid treatment, symptoms improved in 44 of 45 patients (97.8%). Among the 29 patients with clear follow-up information on ACTH and cortisol, 2 cases (6.9%) reported partial recovery of the ACTH/cortisol axis, 27 patients (93.1%) had no documented recovery, and no clear record of complete recovery was observed. Among the 43 patients with vital status data, 39 cases (90.7%) were alive at the last follow-up, and 4 cases (9.3%) died. Because information on cause of death, tumor progression, and the potential relationship with pituitary toxicity was incomplete, we did not attempt to attribute the reported deaths to pituitary toxicity.

## 4 Discussion

This pooled analysis summarized the clinical characteristics, treatment patterns, and outcomes of 45 patients with nivolumab-associated pituitary toxicity identified from 43 published reports. Male patients accounted for 73.3% of the published cases in this pooled analysis. A male predominance has also been described in previous reports of ICI-related hypophysitis (6, 13, 16); however, because the present study was based on published case reports without a comparison group, this observation should be considered descriptive and hypothesis-generating rather than evidence of a sex-related susceptibility. More than half of the published cases originated from Japan. This geographic clustering may reflect differences in reporting practices, clinical awareness, publication patterns, or healthcare settings. In the absence of population-based denominator data, it should not be interpreted as evidence of increased susceptibility in any geographic or ethnic population.

This case summary of 45 patients with nivolumab-associated pituitary toxicity identified a clinically recognizable but low-certainty pattern: patients commonly presented with nonspecific symptoms, including fatigue, anorexia, nausea/vomiting, hypotension, and hyponatremia, and were subsequently found to have abnormalities of the ACTH–cortisol axis. However, most patients with evaluable pituitary MRI findings had no definite structural abnormalities. This pattern is consistent with previous observations of PD-1/PD-L1-associated pituitary toxicity, which often differs from the “classic hypophysitis” phenotype associated with CTLA-4 inhibitors and characterized by headache, pituitary enlargement, and involvement of multiple pituitary axes (10, 12, 13). This difference may be related to the different pathogenesis of the two classes of immune checkpoint inhibitors. Iwama et al. found that CTLA-4 is expressed in pituitary cells, and anti-CTLA-4 antibodies may directly damage pituitary tissue through type II hypersensitivity (complement dependent cytotoxicity), leading to diffuse inflammation and multiple axis dysfunction (12). Compared with CTLA-4 inhibitor-associated hypophysitis, anti-PD-1/PD-L1-related pituitary toxicity more commonly presents with ACTH–cortisol axis involvement, whereas headache, multiple pituitary-axis involvement, and pituitary MRI abnormalities are relatively uncommon (13, 14, 17). The exact pathogenic mechanism remains to be further elucidated. Faje et al. also described anti-PD-1-associated hypophysitis as a clinical entity distinct from ipilimumab-associated hypophysitis (13). Importantly, the term “pituitary toxicity” in the present study encompasses several related but non-identical clinical phenotypes, including hypophysitis, IAD, and central/secondary adrenal insufficiency. Their aggregation reflects the heterogeneous and sometimes overlapping terminology used in published case reports and should not be interpreted as indicating that these entities are diagnostically equivalent. Accordingly, the pooled cohort should be interpreted as a collection of heterogeneous, author-attributed pituitary–adrenal events rather than a uniform cohort of confirmed hypophysitis.

In this study, hyponatremia occurred in 72.7% of evaluable cases and, together with fatigue, anorexia, nausea/vomiting, and hypotension, represented an important clinical clue to nivolumab-associated pituitary toxicity. Glucocorticoid deficiency can impair free-water excretion by reducing the suppression of arginine vasopressin and decreasing renal free-water clearance, thereby resulting in or exacerbating dilutional hyponatremia (18). However, hyponatremia in patients with cancer is multifactorial and may also be associated with reduced oral intake, vomiting, infection, diuretic use, syndrome of inappropriate antidiuretic hormone secretion, renal dysfunction, or concomitant medications (19, 20). Therefore, hyponatremia alone cannot establish the diagnosis of pituitary toxicity. In patients receiving nivolumab who develop progressive or otherwise unexplained hyponatremia, clinicians should assess compatible symptoms together with morning cortisol and ACTH concentrations, blood glucose, hemodynamic status, and other relevant endocrine parameters. Previous case series have suggested that a gradual decline in serum sodium may precede the recognition of isolated ACTH deficiency during anti-PD-1 therapy (21, 22). Nevertheless, the currently available evidence is insufficient to regard hyponatremia as an independent predictor of pituitary toxicity. Serum sodium abnormalities may serve as a nonspecific clinical clue that prompts evaluation of the ACTH–cortisol axis in symptomatic patients receiving nivolumab, but the present data do not establish hyponatremia as a predictor of pituitary toxicity.

Eight patients (17.8%) developed pituitary toxicity after nivolumab discontinuation, with delays of up to 56 weeks. Previous case reports have also described hypophysitis or secondary adrenal insufficiency several months after nivolumab discontinuation (23, 24). Although these observations do not establish the frequency or predictors of delayed toxicity, they indicate that nivolumab-associated pituitary toxicity may be recognized after treatment cessation and support continued clinical awareness when compatible symptoms occur. Postow et al. noted that irAEs may occur weeks to months after ICI discontinuation, supporting the need for continued clinical vigilance beyond the active treatment period (5).

Among the 29 patients with available hormonal follow-up, only 2 (6.9%) had documented partial recovery of the ACTH–cortisol axis, while no complete recovery was reported. This observation is consistent with previous reports suggesting that ACTH deficiency after immune checkpoint inhibitor therapy may be prolonged or persistent (25). However, the present estimate should be interpreted cautiously because hormonal follow-up was unavailable for a substantial proportion of patients and follow-up duration varied across reports. Therefore, recovery may have been underestimated, particularly in patients with limited or short-term follow-up. Long-term endocrine reassessment remains important to determine whether recovery occurs over time (26).

In terms of treatment, hydrocortisone was the most commonly used glucocorticoid in this study. Hydrocortisone was the most commonly reported glucocorticoid for both initial treatment (74.4%) and maintenance replacement therapy (82.1%), consistent with recommendations from the ASCO and ESMO guidelines (26, 27). For patients with stable ACTH deficiency or secondary adrenal insufficiency, hydrocortisone at a dose of 15 to 25 mg per day is usually administered orally in 2 to 3 doses for physiological replacement. When there is an adrenal crisis or hemodynamic instability, intravenous stress doses of glucocorticoids should be administered promptly (26, 28).

Based on the clinical patterns observed in the present pooled analysis and current endocrine management principles, we summarized a practical clinical approach for suspected nivolumab-associated pituitary toxicity (**Figure 2**). Patients receiving or previously exposed to nivolumab who develop compatible nonspecific symptoms, particularly when accompanied by unexplained hyponatremia or hypotension, should undergo prompt assessment of cortisol, ACTH, electrolytes, and other relevant endocrine parameters. Pituitary MRI may assist diagnostic evaluation in selected patients but should not be used to exclude the diagnosis because structural abnormalities are frequently absent. When adrenal crisis or hemodynamic instability is suspected, glucocorticoid treatment should not be delayed for completion of endocrine testing or imaging. Long-term management should include appropriate hormone replacement, education regarding stress dosing, and longitudinal endocrine reassessment.

**Figure 2:**
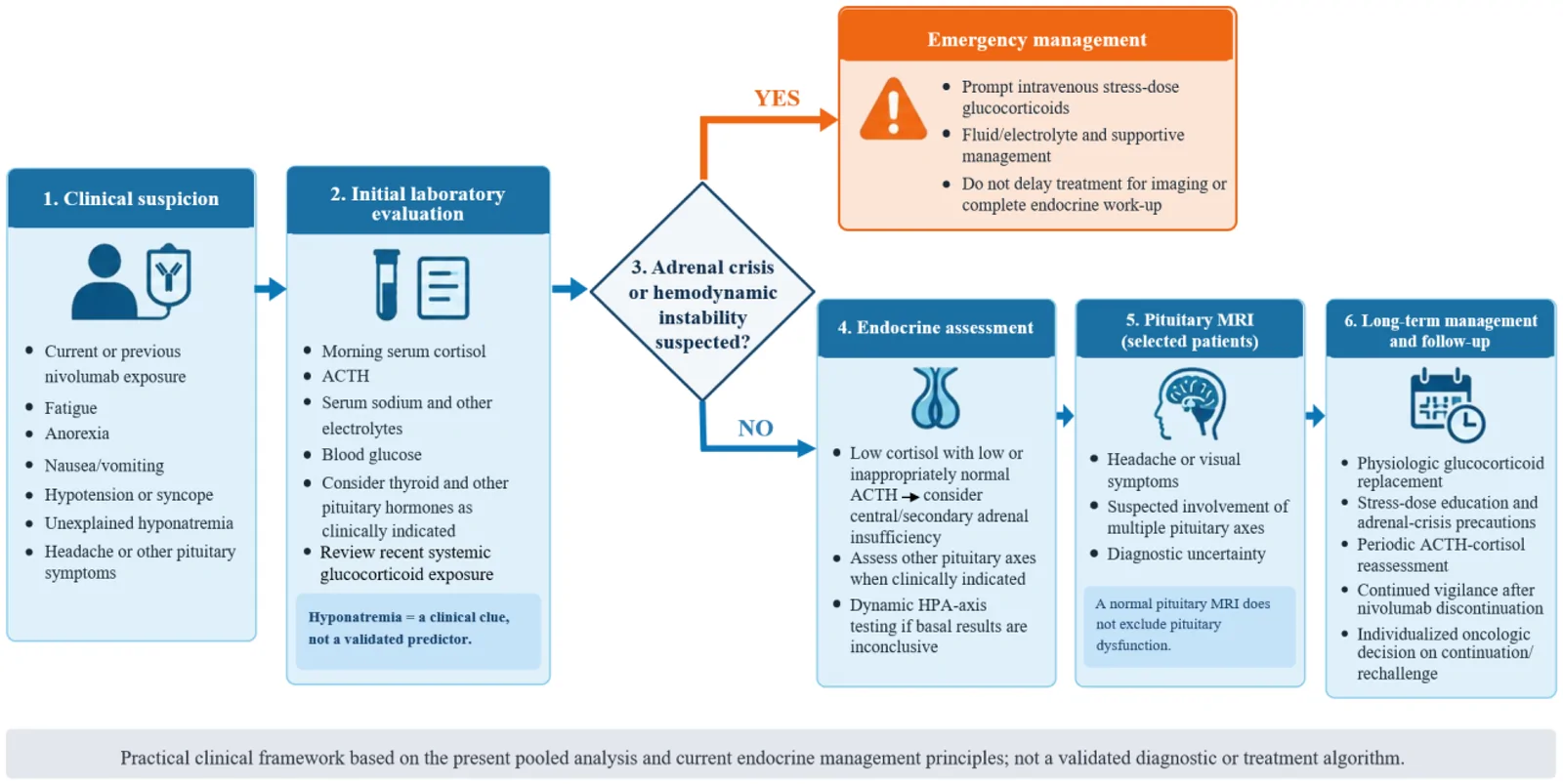
Practical clinical approach to suspected nivolumab-associated pituitary toxicity. The algorithm integrates clinical features observed in the present pooled analysis with general principles of endocrine evaluation and management from current clinical guidelines. It is intended as a practical clinical framework rather than a validated diagnostic or treatment algorithm. Recent systemic glucocorticoid exposure should be considered because it may suppress ACTH and cortisol and confound the assessment of central adrenal insufficiency. A normal pituitary MRI does not exclude nivolumab-associated pituitary–adrenal dysfunction, and urgent glucocorticoid treatment should not be delayed when adrenal crisis is suspected.

Six patients were rechallenged with nivolumab, and no recurrence of pituitary toxicity was reported. However, the number of rechallenged patients was very small, and the definitions of rechallenge, follow-up duration, oncologic context, and hormone replacement strategies varied across reports. Therefore, limited case-level evidence suggests that nivolumab rechallenge may be feasible in selected patients receiving adequate hormone replacement, although larger datasets are required to better define its safety. Current guidelines also indicate that endocrine immune-related adverse events do not necessarily require permanent discontinuation of immune checkpoint inhibitor therapy once appropriate hormone replacement has been established (26).

## 5 limitations

This study has several limitations. First, the evidence base consisted entirely of case reports and small case series, which are inherently susceptible to publication, selection, and reporting biases. No formal methodological quality or risk-of-bias assessment was performed because this study was designed as a descriptive pooled analysis of individual published cases rather than a comparative or effect-estimating systematic review. Nevertheless, the absence of a standardized critical appraisal remains a limitation, and the findings should therefore be interpreted as descriptive and hypothesis-generating rather than as quantitative estimates of risk or causal associations. Second, there was a lack of uniformity across studies in diagnostic criteria, hormone assay units, reference ranges, dynamic testing protocols, timing of MRI, glucocorticoid dosages, and definitions of follow-up, which limited the comparability of the findings. Another important limitation is that previous or concomitant systemic glucocorticoid exposure before ACTH and cortisol assessment was not systematically captured in the pooled dataset. In oncology patients, exogenous glucocorticoids may suppress both ACTH and cortisol and thereby mimic central adrenal insufficiency. Therefore, diagnostic misclassification cannot be excluded in some cases, particularly when isolated ACTH–cortisol axis abnormalities were reported without additional pituitary hormone deficiencies or supportive pituitary MRI findings. This uncertainty should be considered when attributing pituitary–adrenal dysfunction to nivolumab. Third, 57.8% of the cases came from Japan; while this may reflect regional reporting practices, healthcare-seeking pathways, or population characteristics, it should not be directly interpreted as evidence of increased susceptibility in any particular ethnic group. Fourth, certain variables were missing, especially regarding multi-pituitary axis assessment, hormone recovery, treatment efficacy, and attribution of death. Fifth, no control group was included, precluding comparisons of risk among different drugs, regimens, or populations. Going forward, the establishment of a multicenter prospective registry that standardizes baseline and follow-up measurements of ACTH, cortisol, and serum sodium, defines clear MRI indications, and unifies glucocorticoid protocols and recovery criteria would help generate more robust evidence to inform clinical decision-making.

## 6 Conclusion

The pooled cases represent heterogeneous author-attributed pituitary–adrenal events rather than uniformly confirmed hypophysitis. Nivolumab-associated pituitary toxicity most commonly involves dysfunction of the ACTH–cortisol axis and often presents with nonspecific manifestations, including fatigue, anorexia, nausea/vomiting, hypotension, and hyponatremia, while pituitary MRI may remain unremarkable. Importantly, pituitary toxicity may occur not only during active nivolumab treatment but also after treatment discontinuation, with delayed onset reported several weeks to months after the last dose. Therefore, new or progressive compatible symptoms and unexplained hyponatremia after nivolumab withdrawal should still prompt timely assessment of cortisol, ACTH, serum electrolytes, and hemodynamic status, with adrenal crisis promptly excluded and managed when suspected. Case-level data suggest that symptoms generally improve after glucocorticoid replacement therapy, whereas recovery of the ACTH–cortisol axis appeared uncommon among patients with available follow-up, although incomplete and heterogeneous follow-up may have led to underestimation of recovery. Limited case-level evidence suggests that continuation or rechallenge with nivolumab may be feasible in selected patients receiving appropriate hormone replacement, but this observation requires confirmation in larger datasets. These findings are intended to improve clinical awareness of both on-treatment and delayed presentations of nivolumab-associated pituitary toxicity; they cannot replace prospective evidence or individualized guideline-based decision-making.

## Data Availability

The raw data supporting the conclusions of this article will be made available by the authors, without undue reservation.
