# Supplementary material for: Clinical characteristics, management, and outcomes of nivolumab-associated pituitary toxicity: a pooled analysis of published case reports and case series
Source: Front Endocrinol (Lausanne). 2026 Oct 21;17:1929591. doi: 10.3389/fendo.2026.1929591 (PMC13730941; doi:10.3389/fendo.2026.1929591)
Supplement: Supplementary file 1 [file Table1.docx]

**Supplementary Table S1. Database-specific search strategies**

| **Database** | **Search strategy** | **Records** |
| --- | --- | --- |
| Web of Science Core Collection | TS=(nivolumab OR Opdivo OR "BMS-936558") AND TS=(hypophysitis OR hypopituitarism OR "isolated ACTH deficiency" OR "ACTH deficiency" OR "secondary adrenal insufficiency" OR "central adrenal insufficiency" OR "pituitary dysfunction") | 369 |
| PubMed | (nivolumab[Title/Abstract] OR Opdivo[Title/Abstract] OR "BMS-936558"[Title/Abstract]) AND (hypophysitis[Title/Abstract] OR hypopituitarism[Title/Abstract] OR "isolated ACTH deficiency"[Title/Abstract] OR "ACTH deficiency"[Title/Abstract] OR "secondary adrenal insufficiency"[Title/Abstract] OR "central adrenal insufficiency"[Title/Abstract] OR "pituitary dysfunction"[Title/Abstract]) | 259 |
| Embase | (nivolumab OR Opdivo OR "BMS-936558") AND (hypophysitis OR hypopituitarism OR "isolated ACTH deficiency" OR "ACTH deficiency" OR "secondary adrenal insufficiency" OR "central adrenal insufficiency" OR "pituitary dysfunction") | 1949 |
| CNKI | SU=(纳武利尤单抗 + 纳武单抗 + 尼沃单抗 + 尼沃利尤单抗 + 欧狄沃) AND SU=(垂体炎 + 垂体功能减退 + 垂体功能不全 + 孤立性ACTH缺乏 + ACTH缺乏 + 促肾上腺皮质激素缺乏 + 继发性肾上腺皮质功能不全 + 中枢性肾上腺皮质功能不全) | 4 |
| Wanfang Data | (纳武利尤单抗 OR 纳武单抗 OR 尼沃单抗 OR 尼沃利尤单抗 OR 欧狄沃) AND (垂体炎 OR 垂体功能减退 OR 垂体功能不全 OR 孤立性ACTH缺乏 OR ACTH缺乏 OR 促肾上腺皮质激素缺乏 OR 继发性肾上腺皮质功能不全 OR 中枢性肾上腺皮质功能不全) | 11 |
